# The Predictive Value of Interim and Final [18F] Fluorodeoxyglucose Positron Emission Tomography after Rituximab-Chemotherapy in the Treatment of Non-Hodgkin's Lymphoma: A Meta-Analysis

**DOI:** 10.1155/2013/275805

**Published:** 2013-08-14

**Authors:** Yuyuan Zhu, Jianda Lu, Xin Wei, Shaoli Song, Gang Huang

**Affiliations:** ^1^Department of Nuclear Medicine, Renji Hospital, Jiaotong University, Shanghai 200127, China; ^2^Department of Nuclear Medicine, Renji Hospital, Shanghai Jiaotong University School of Medicine, Shanghai 200127, China; ^3^Renji Hospital, School of Medicine, Shanghai Jiaotong University, Shanghai 200127, China

## Abstract

*Background and Purpose*. The aim of this study is to determine the prognostic value of interim and final FDG-PET in major histotypes of B-cell NHL patients treated with rituximab containing-chemotherapy. *Methods*. We searched for articles published in English, limited to lymphoma, rituximab, and FDG-PET, and dedicated to deal with the impact on progression and survival. The log hazard ratios (HR) and their variances were estimated. *Results*. A PubMed and Scopus review of published trials identified 13 studies of Progression-free survival (PFS) and overall survival (OS) which were set as the main outcome measures. The combined HRs of I-PET for PFS and OS in DLBCL were 4.4 (*P* = 0.11) and 3.99 (*P* = 0.46), respectively. The combined HRs of F-PET for PFS and OS in DLBCL were 5.91 (*P* = 0.39) and 6.75 (*P* = 0.92), respectively. Regarding to non-DLBCL with F-PET, the combined HRs of F-PET for PFS and OS were 4.05 (*P* = 0.79) and 5.1 (*P* = 0.51), respectively. No publication bias existed. *Conclusion*. In DLBCL, both I-PET and F-PET can be performed for survival and progression analysis. But in other B-cell subtypes such as follicular lymphoma (FL) and mantle cell lymphoma (MCL), it would be necessary to perform F-PET for predictive purposes.

## 1. Introduction 

The use of [18F] fluorodeoxyglucose positron emission tomography (^18^F-FDG PET) imaging in the management of lymphoma has remarkably expanded after the realization of the metabolic features of lymphoma cells [1, 2]. PET/CT imaging provides both anatomic and functional information which is fundamentally altering staging, guiding the choice of treatment modality, response monitoring, and response assessment for lymphomas. Meanwhile, it can provide useful information concerning prognosis for the risk stratified therapy. The application of interim FDG-PET in the risk stratification of Hodgkin's lymphoma is very successful [3]. But the benefits of FDG-PET/CT in the management of NHL are uncertain. Previous meta-analysis about the prognostic value of PET in Hodgkin's lymphoma or non-Hodgkin's Lymphoma showed no consistent conclusions due to the heterogeneity caused by different study populations, variations of imaging condition, inconsistent imaging interpretation criteria, and lack of uniformed treatment regimens [4]. All these factors impact on the PET results which may coinstantaneousy influence the management of the progression and survival of lymphoma patients in most clinical situations. NHL is a heterogeneous group of tumors with different aggressiveness. Subtypes like diffuse large B-cell lymphoma, follicular lymphoma, and mantle cell lymphoma are all FDG-avid [5], so that FDG-PET could be a potential prognostic imaging modality for survival prediction. Therefore, we, through the literature review, performed a meta-analysis concentrating on interim and final FDG-PET in major histological subtypes of B-cell NHL patients (including DLBCL and non-DLBCL) treated with first-line rituximab containing-chemotherapy to assess the prognostic value of PET.

## 2. Materials and Methods 

### 2.1. Literature Search

Studies were identified by a comprehensive electronic literature search [6] of abstracts of studies assessing the predictive value of PET for the human lymphoma. We conducted a search on the MEDLINE and Scopus databases, using keywords (PET, positron emission tomography, or SUV), lymphoma (rituximab, R-CHOP, or R), humans, and English. 

### 2.2. Selection of Studies

Four investigators, including three physicians and one biostatistician, reviewed each publication independently and scored them according to a quality scale as described in the appendix. Each item was graded with a value between 0 and 2. This quality scale evaluated several dimensions of the methodology, grouped into four main categories: the scientific design, the generalization of the results, the analysis of the study data, and the PET reports. This quality scale was modified on the basis of the European lung cancer working party quality scale for biological prognostic factors for lung cancer introduced by Steels et al. [20]. To assess the PET reports, the scoring items previously introduced by Berghmans et al. [21] were used. The scores were compared and a consensus value for each item was reached in meetings at which at least two-thirds of the investigators needed to be present. 

The participation of many readers was a guarantee for the correct interpretation of the articles. As the scores were objective, a consensus was always obtainable. The final scores were expressed as percentages, with higher values reflecting a better methodological quality. Each category had a maximum score of 10 points; hence, the overall maximum score was 40 points. Two reviewers independently assessed the quality items, and discrepancies were resolved by consensus. When an item was not applicable in a study, the theoretically attributable points were not taken into account in the total of the concerned category. 

The studies about NHL patients mainly treated with rituximab-regimen plus CHOP (cyclophosphamide, doxorubicin, vincristine, and prednisone) or CHOP-like intensive chemotherapy monitored by FDG-PET providing survival data for the meta-analysis were potential for full-text evaluation. Only the studies reporting or providing data to make univariate analysis or results for survival were considered for the aggregation of the survival data. 

Detailed inclusion criteria are as follows:

1. Including more than 10 patients with histologically proven NHL patients treated with first-line R-chemo regimen with or without proceeding treatment such as radioimmunotherapy (RIT), BEAM chemotherapy (carmustine, etoposide, cytarabine, and melphalan regimen), and autologous stem cell transplantation (ASCT).
2. Use interim and/or final PET to monitor therapy response and predict the survival of lymphoma patients.
3. Use positive and negative results of FDG-PET as a predicting factor according to SUV cutoff value or visual analysis.
4. Survival data of hazard ratio was extractable.
5. Treatment of lymphoma is not risk-adapted by the result of FDG-PET.

### 2.3. Statistical Methods

Survival data from each study were analyzed in terms of the Kaplan-Meier curves, unless hazard ratios (HRs) were reported, and compared to calculate HR and 95% confidence intervals (CI) as previously described by Parmar et al. [22] and Tierney et al. [23]. In brief, effects were measured from the observed minus expected difference (O−), and variance (V) was generated using the reported summary statistics, by the one step approximation exp [(O−)/V]. These effects were combined to estimate the overall (pooled) effect of the PET-positive versus PET-negative arm. An HR < 1 denotes the survival benefit from a positive PET scan, whereas an HR > 1 indicates an increased risk of progression and death. 

Statistical heterogeneity was measured using the chi-squared *Q* test (*P* < 0.10 was considered to represent significant statistical heterogeneity) and the *I* ^2^ statistic, as described by Higgins et al. [24]. Subgroup analysis was performed if heterogeneity existed. Publication bias including funnel plot and Egger's test was performed.

Survival rates on the graphical representation of the survival curves were read by Engauge Digitizer version 2.5. HRs and their variations were calculated by STATA version 12.0 and Review Manager 5.2.0. 

## 3. Results 

### 3.1. Study Selection and Characteristics Analysis

**Figure 1:**
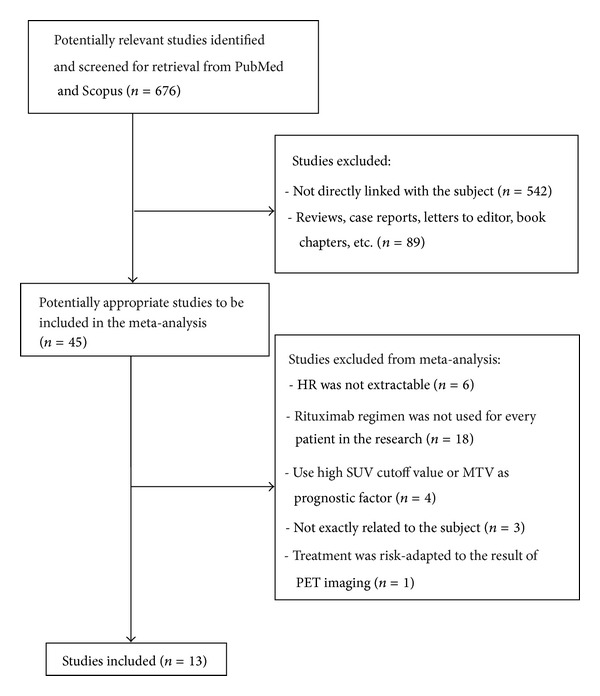
The study selection process (HR: hazard ratios; SUV: standardized uptake value).

The detailed study selection process was described in Figure 1. The electronic searches yield 676 potentially eligible articles from all databases. Of all these articles, 45 were analyzed. Thirty two of these studies were excluded because of the following: unable to calculate the log HR and its variance (*n* = 6), not using rituximab regimen in every patient of the study (*n* = 18), using a relatively high SUV cutoff or MTV as a prognostic factor (*n* = 4), not exactly related to the research subject (*n* = 3), and its treatment being risk-adapted to the result of PET (*n* = 1) [25]. Finally, a total of 13 studies (all in English, 8 retrospective and 5 prospective) [7–19] were used for the analysis. 

The principal characteristics of the 13 studies evaluated for the meta-analysis were described in Table 1. A total of 1160 patients, with a predominance of male DLBCL patients, were included in this prognostic meta-analysis. About half of the patients were graded intermediate or high IPI score. The median follow-up period ranged from 20 to 38 months. Seven studies [8–10, 12, 13, 16, 17] used FDG-PET/CT, and six studies [7, 11, 14, 15, 18, 19] used FDG-PET. Nine [7, 9–11, 13, 16–19] of these studies achieved definite statistical significance, while other four showed undetermined results [8, 12, 14, 15]. Ten studies included a single histotype of NHL [8–13, 16–19] and three studies [7, 14, 15] included a mixed subtype of NHL with a majority of DLBCL. In order to ensure enough included articles, the latter three were categorized into DLBCL subgroups for pooling data instead of being excluded. Meta-analysis was performed based on each lymphoma subtype, for the clinical interpretation of FDG-PET is usually on the basis of patient diagnosis. As I-PET is not routinely performed in non-DLBCL patients [26], and few existing researches about I-PET showed a positive predictive value in non-DLBCL patients [17, 19], only I-PET and F-PET in patients with DLBCL and F-PET in non-DLBCL were evaluated separately (Table 1). 

In a majority of DLBCL patients, nine studies dealt with the prognostic value of I-PET which was performed after 2–4 cycles of R-chemotherapy [7–9, 11, 12, 14–16, 18], in which 9 studies presented an extractable HR value for PFS (progression-free survival) and 8 studies for OS (overall survival) (Table 1). Four studies dealt with the prognostic value of F-PET which was performed after the 6–8 cycles of R-chemotherapy [12, 14–16], in which 4 studies presented an extractable HR value for PFS and 3 studies for OS. In non-DLBCL patients, four studies dealt with the prognostic value of F-PET [10, 13, 17, 19], in which 4 studies presented an extractable HR value for PFS and 3 studies for OS (Table 1). On the whole, approximately 34 HRs were extracted, of which 8 HR values and their confidence intervals were directly from the articles, whereas the other 26 HRs were extracted from the K-M curves. Six meta-analyses were performed for both OS and PFS of I-PET and F-PET in NHL patients afterwards. One study by Le Dortz et al. [13] concerning the response monitor of follicular lymphoma combined I-PET and F-PET together with a majority of final data, and it was categorized into the final group. 

### 3.2. Quality Assessment

Overall, the global quality score ranged from 50 to 89%, with a median score of 72.3% (Table 1). An attempt was made to contact the authors, if necessary, to obtain missing details of the methodological quality. 

### 3.3. Meta-Analysis

**Figure 2:**
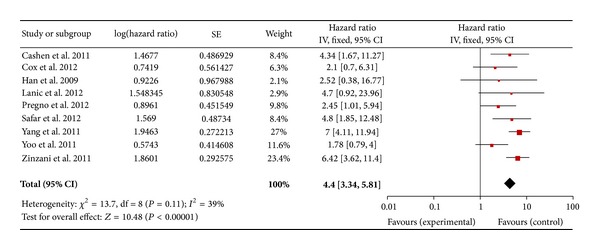
Forest plot of nine included studies in DLBCL (I-PET PFS). Pooled effect (HR) and heterogeneity test of an I-PET-positive scan on PFS in DLBCL patients (PET: positron emission tomography; PFS: progression-free survival; HR: hazard ratio; DLBCL: diffuse large B-cell lymphoma).

**Figure 3:**
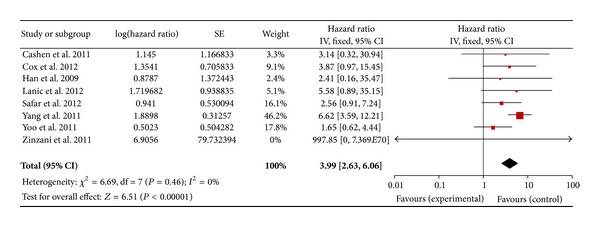
Forest plot of eight included studies in DLBCL (I-PET, OS). Pooled effect (HR) and heterogeneity test of an I-PET-positive scan on OS in DLBCL patients (PET: positron emission tomography; OS: overall survival; HR: hazard ratio; DLBCL: diffuse large B-cell lymphoma).

**Figure 4:**
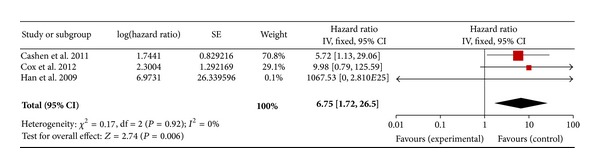
Forest plot of three included studies in DLBCL (F-PET, PFS). Pooled effect (HR) and heterogeneity test of an F-PET-positive scan on PFS in DLBCL patients (PET: positron emission tomography; PFS: progression-free survival; HR: hazard ratio; DLBCL: diffuse large B-cell lymphoma).

**Figure 5:**
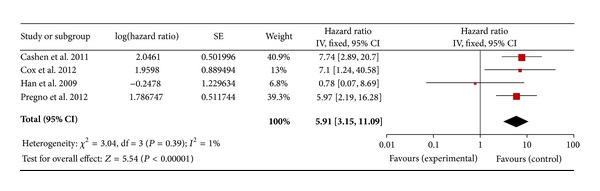
Forest plot of four included studies in DLBCL (F-PET, OS). Pooled effect (HR) and heterogeneity test of an F-PET-positive scan on OS in a majority of DLBCL patients (PET: positron emission tomography; OS: overall survival; HR: hazard ratio; DLBCL: diffuse large B-cell lymphoma).

**Figure 6:**
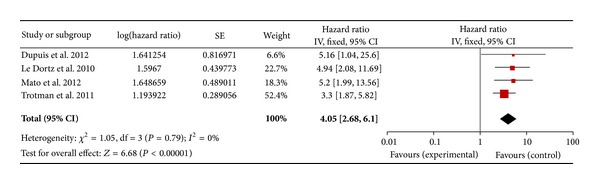
Forest plot of four included studies in non-DLBCL (F-PET, PFS). Pooled effect (HR) and heterogeneity test of F-PET-positive scan on PFS in non-DLBCL patients (PET: positron emission tomography; PFS: progression-free survival; HR: hazard ratio; DLBCL: diffuse large B-cell lymphoma).

Regarding the DLBCL with an I-PET, 9 studies for PFS and 8 studies for OS were included. In a fixed effect model, the combined HRs of I-PET for PFS and OS were 4.4 (95% CI: 3.34–5.81, *P* = 0.11) (Figure 2) and 3.99 (95% CI: 2.63–6.06, *P* = 0.46) (Figure 3), respectively. Regarding the DLBCL with F-PET, there were 4 studies for PFS and 3 studies for OS. The combined HRs of F-PET for PFS and OS were 5.91 (95% CI: 3.15–11.09, *P* = 0.39) (Figure 4) and 6.75 (95% CI: 1.72–26.50, *P* = 0.92) (Figure 5), respectively. Regarding the non-DLBCL with F-PET, 4 studies for PFS and 3 studies for OS were included. The combined HRs of F-PET for PFS and OS were 4.05 (95% CI: 2.68–6.1, *P* = 0.79) (Figure 6) and 5.1 (95% CI: 2.54–10.23, *P* = 0.51) (see Figure S3 in supplementary material available online at http://dx.doi.org/10.1155/2013/275805). All pooling data were statistically homogeneous. Meaning that for both I-PET and F-PET in DLBCL patients, a positive PET scan indicated a worse survival prognosis and a higher risk of progression than a negative PET scan, and in non-DLBCL patients, a positive F-PET could be predictive of more recurrence and worse survival.

### 3.4. Publication Bias for HR of I-PET in DLBCL Patients

The evaluation of publication bias showed that Egger's test results for PFS and OS were both insignificant (*P* = 0.119, *P* = 0.485). The funnel plots for publication bias of I-PET for PFS and OS (Figures S1 and S2) show little asymmetry. These results indicated no publication bias for the HR pooling of I-PET in DLBCL patients for either PFS or OS. 

## 4. Discussion 

As one of the ten leading cancer types in both men and women, non-Hodgkin's Lymphoma caused 70,130 estimated new cancer cases and 18,940 estimated deaths in the USA during the year of 2012 [27]. The combination of the anti-CD20 monoclonal antibody rituximab (R) with the standard doses of chemotherapy has dramatically improved the clinical outcomes of NHL patients. Nevertheless, significant proportions of patients show disease progression or relapse after a good initial response [28, 29]. These patients may require alternative approaches, such as early intensive chemotherapy followed by ASCT or participation in clinical trials of new molecular targeted agents. It is essential to identify these patients as early as possible, so that they can be switched to other treatments for a longer survival. 

Consequently, finding reliable prognostic indicators would be very helpful in the management of NHL patients. The most commonly used factors are histopathological subtypes and the International Prognostic Index (IPI). The previousy used IPI for aggressive lymphoma was developed specifically to stratify NHL patients for overall survival, but it may not be reliable for patients with different outcomes from the same IPI group [7, 30]. Other than that, it was suggested that I-PET or F-PET, immunephenotypes, and gene expressions could also be additional predictive factors [19, 31–33]. 

Based on the statistical analysis of a total of 1160 NHL patients, with a predominance of male DLBCL patients, our study confirms the independent prognostic value of FDG-PET in NHL patients treated with first-line R-chemotherapy. I-PET and F-PET in DLBCL and F-PET in non-DLBCL are all independent prognostic factors for survival and recurrence without statistical heterogeneity. 

NHL consists of approximately 80% of B-cell lymphoma cases, and the remaining 20% are of T-cell and natural killer (NK) cell origin [5]. Most CD20+ B-cell lymphomas are suggested for R-chemotherapy if clinically available. Though FDG-PET has an excellent accuracy in baseline detection in cases of diffuse large B-cell lymphoma, follicular lymphoma, and mantle cell lymphoma [34], the FDG uptake of B-cell lymphoma varies according to diverse histotypes and aggressiveness and so does the predictive value of ^18^F-FDG PET. The baseline FDG uptake of DLBCL is much higher which makes the visual and semiquantitative interpretation of the SUV percentage change more sensitive. While the mean uptakes of FL and MCL are relatively lower [35], the lymphoma subtypes could be a major potential source of heterogeneity to the predictive value of FDG-PET suggested in the previous meta-analysis.

Therefore, in patients with DLBCL, I-PET and F-PET should be performed for the prognosis evaluation and risk stratification. That would be more valuable for the management of DLBCL patients. As for patients with other subtypes of NHL such as FL and MCL, it would be necessary to perform final FDG-PET. 

There are several limitations of our meta-analysis. First, only published articles were included, and the articles were restricted to the articles published in English. Second, studies with statistically significant results were more often published, whereas those with no statistically significant results were not. Third, even though they were published, more often than not, they were not assessable because of the more concise reports of results. These reasons may have led to the publication bias found in the present paper. Fourth, most of the HRs were extrapolated from the survival curves. Although three readers independently read the survival rates on the graphical representation of the survival curves, the strategy could not ensure a complete accuracy in the extracted survival rates. Fifth, studies included were retrospective and we suggest that larger prospective, high-quality, and multicenter studies should be conducted according to different histological subtypes of NHL especially in NHL subtypes other than DLBCL. In conclusion, further studies of cost-effectiveness analysis should be conducted with regard to the techniques predicting the survival of B-cell NHL.

## Supplementary Material

Supplementary material included 2 funnel graphs for potential publication bias in studies of I-FDG-PET on PFS( Figure S1) and OS (Figure S2) in DLBCL patients, and 1 forest plot(Figure S3) of four included studies in non-DLBCL (F-PET, OS).(PET: positron emission tomography, PFS: progression free survival, OS: overall survival, HR: hazard ratio; DLBCL: diffuse large B-cell lymphoma).

## Figures and Tables

**Table 1:** Characteristics of the 13 studies included in the meta-analysis.

Study	Publication year	No. of NHL patients	Study design	Stage	Functional imaging	PET timing	Survival data	PET (+) as prognostic factor for survival	Methodology score (%)
Zinzani et al. [7]	2011	DLBCL (*n* = 78) PMLBCL (*n* = 13)	Retrospective	Stage II–IV	PET	I-PET	OS & PFS	Significant	65.79%
Yoo et al. [8]	2011	DLBCL (*n* = 155)	Retrospective	Stage I–IV	PET/CT	I-PET, final PET	OS & PFS	Undetermined	78.95%
Yang et al. [9]	2011	DLBCL (*n* = 159)	Prospective	Stage I–IV	PET/CT	I-PET	OS & PFS	Significant	89.47%
Trotman et al. [10]	2011	FL (*n* = 122)	Prospective	Stage III-IV	PET/CT	F-PET	OS & PFS	Significant	71.05%
Safar et al. [11]	2012	DLBCL (*n* = 112)	Retrospective	Stage III-IV	PET	I-PET	OS & PFS	Significant	71.05%
Pregno et al. [12]	2012	DLBCL (*n* = 88)	Retrospective	Stage I–IV	PET/CT	I-PET, F-PET	PFS	Undetermined	78.95%
Le Dortz et al. [13]	2010	FL (*n* = 45)	Retrospective	Stage I–IV	PET/CT	F-PET	PFS	Significant	71.05%
Han et al. [14]	2009	DLBCL (*n* = 38) MCL (*n* = 13)	Retrospective	Stage I–IV	PET	I-PET, F-PET	OS & PFS	Undetermined	63.16%
Cox et al. [15]	2012	DLBCL (*n* = 73) PMLBCL (*n* = 12)	Prospective	Stage I–IV	PET	I-PET	OS & PFS	Undetermined	76.68%
Cashen et al. [16]	2011	DLBCL (*n* = 50)	Prospective	Stage III-IV	PET/CT	I-PET, F-PET	OS & PFS	Significant	71.05%
Mato et al. [17]	2012	MCL (*n* = 148)	Retrospective	N/A	PET/CT	I-PET, F-PET	OS & PFS	F-PET: significant I-PET: undetermined	50.00%
Lanic et al. [18]	2012	DLBCL (*n* = 57)	Retrospective	N/A	PET	I-PET	OS & PFS	Significant	71.05%
Dupuis et al. [19]	2012	FL (*n* = 111)	Prospective	Grade 1 to 3A	PET	I-PET, F-PET	OS & PFS	Significant (OS of I-PET: undetermined)	84.21%
