# Supplementary material for: The Predictive Value of Interim and Final [18F] Fluorodeoxyglucose Positron Emission Tomography after Rituximab-Chemotherapy in the Treatment of Non-Hodgkin's Lymphoma: A Meta-Analysis
Source: Biomed Res Int. 2013 Aug 14;2013:275805. doi: 10.1155/2013/275805 (PMC3830841; doi:10.1155/2013/275805)
Supplement: Supplementary file 1 — Supplementary material included 2 funnel graphs for potential publication bias in studies of I-FDG-PET on PFS( Figure S1) and OS (Figure S2) in DLBCL patients, and 1 forest plot(Figure S3) of four included studies in non-DLBCL (F-PET, OS).(PET: positron emission tomography, PFS: progression free survival, OS: overall survival, HR: hazard ratio; DLBCL: diffuse large B-cell lymphoma). [file 275805.f1.doc]

**SUPPLEMENTAL INFORMATION**

**The Predictive Value of Interim and Final [18F] fluorinedeoxyglucose - Positron Emission Tomography after Rituximab-chemotherapy in the treatment of Non Hodgkin's Lymphoma: A meta-analysis**

Yuyuan Zhu^1^, Jianda Lu^2^, Xin Wei^2^, Shaoli Song^1^,*Gang Huang^1^

1. Department of Nuclear Medicine, Renji Hospital affiliated to Jiaotong University, Shanghai, China, 200127
2. Renji Hospital, Shanghai Jiaotong University School of Medicine, Shanghai, China, 200127

**Corresponding author**:

*Gang Huang, MD, PhD. Department of Nuclear Medicine, Renji Hospital affiliated to Jiaotong University, Shanghai, China, 200127; Tel.: +86 21 68583530; Fax: +86 21 63842916; E-mail address: huang2802@163.com.

**First authors:**

**Yuyuan Zhu**, MD. Resident of Nuclear Medicine Department, Renji Hospital Shanghai Jiaotong University School of Medicine, Shanghai China. 200127 Tel: +86 18616202507; Fax: +86 21 63842916

E-mail address: hezicase@gmail.com

**Jianda Lu,** Renji Hospital, School of Medicine, Shanghai Jiao tong University, Shanghai China. 200127 Tel: +86 15216715180; Fax: +86 21 63842916; E-mail address: jiandalu@yahoo.cn

Running Title: PET predicts survival of NHL patients treated with R-chemotherapy.

**Table of Contents**

1. **Fig. S1.** Funnel graph for the assessment of potential publication bias in 9 studies of I-FDG-PET on PFS in DLBCL patients (PET: positron emission tomography, PFS: progression free survival)
2. **Fig. S2.** Funnel graph for the assessment of potential publication bias in 8 studies of I-FDG-PET on OS in DLBCL patients (PET: positron emission tomography, OS: overall survival)
3. **Fig. S3.** Forest plot of four included studies in non-DLBCL (F-PET, OS). Pooled effect (HR) and heterogeneity test of F-PET-positive scan on PFS in non-DLBCL patients. (PET : positron emission tomography, PFS: progression free survival; HR: hazard ratio; DLBCL: diffuse large B-cell lymphoma)
4. Appendix

**Fig. S1.**

**
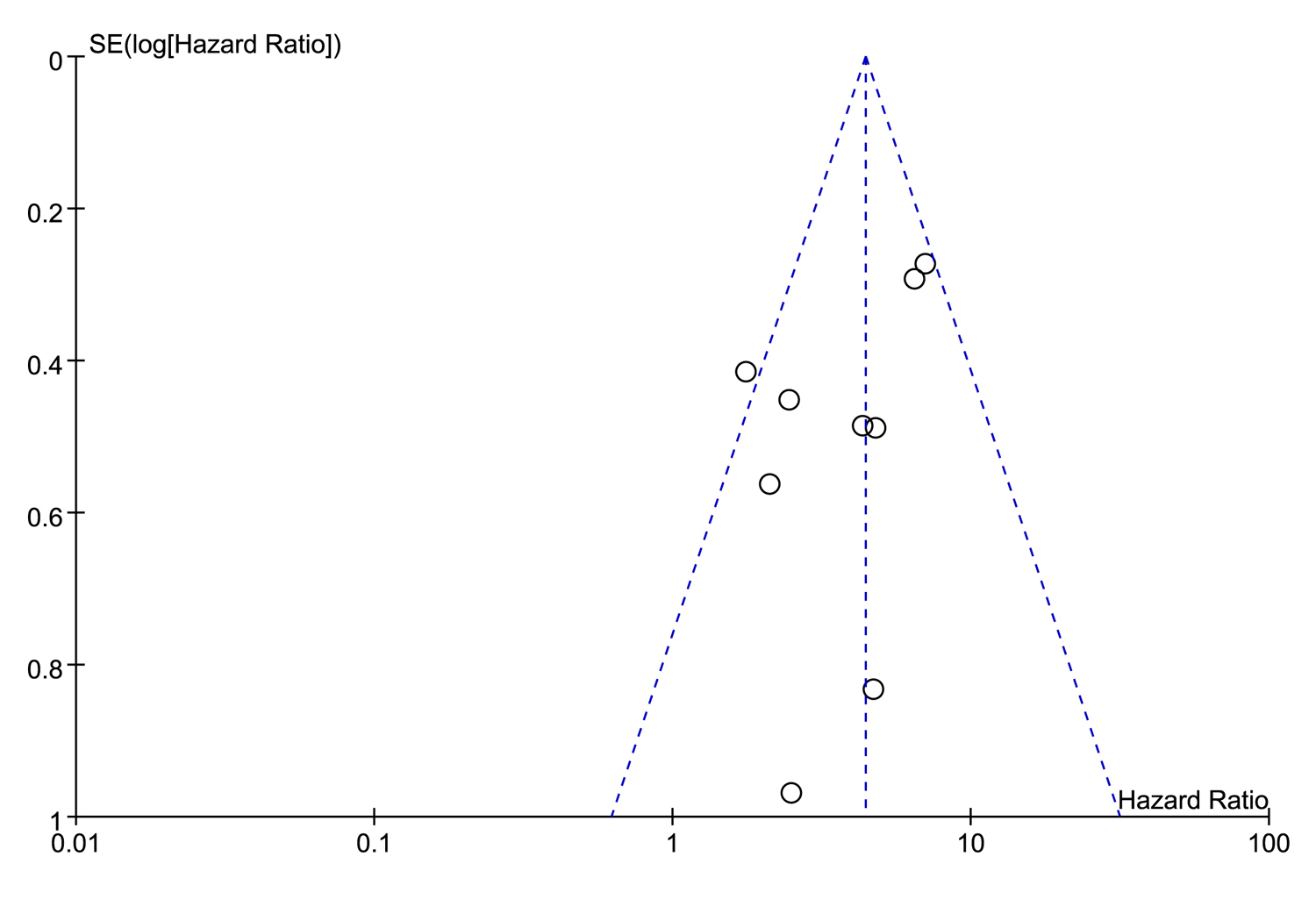
**

**Fig. S2.**

**
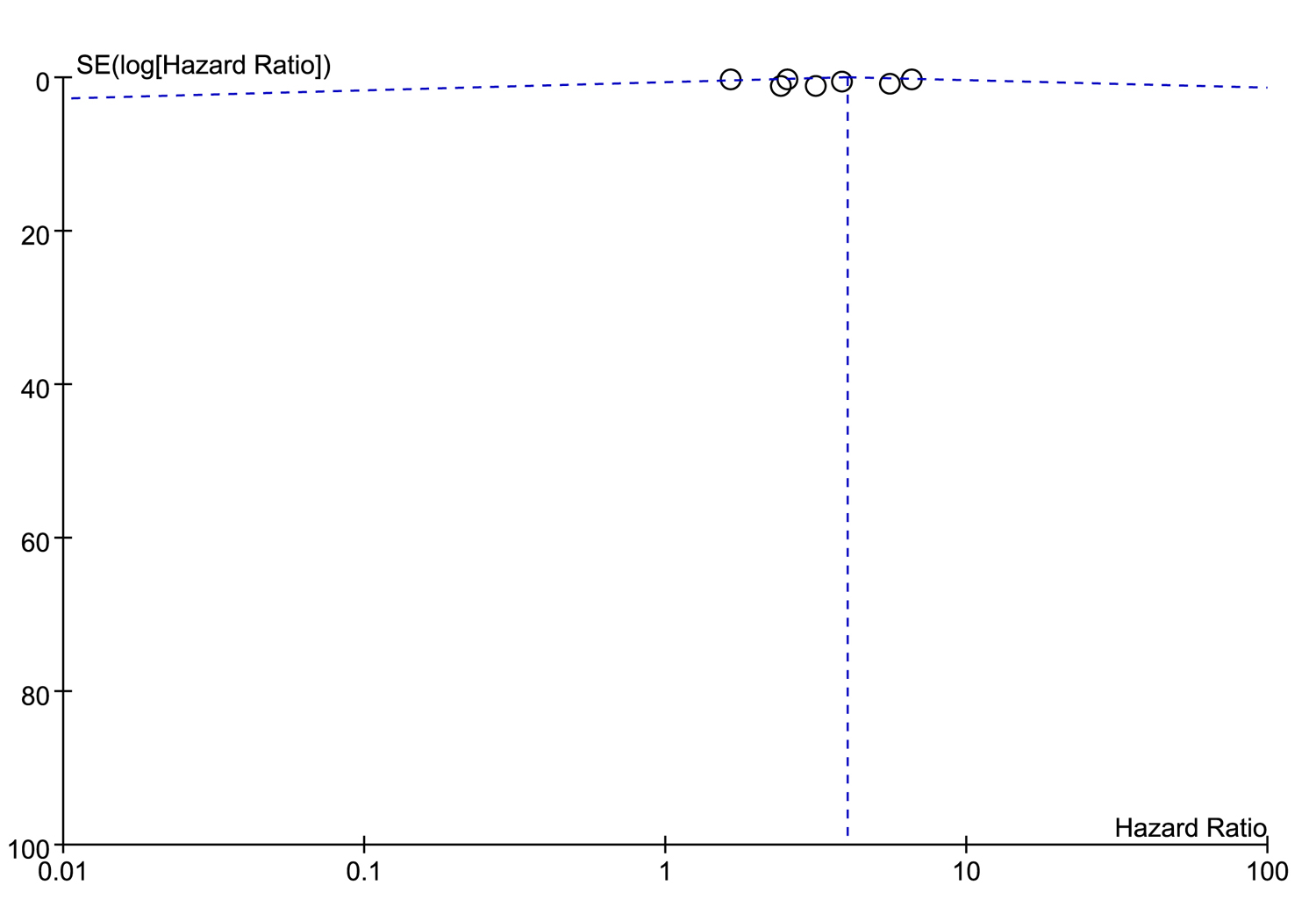
**

**Fig. S3.**

**
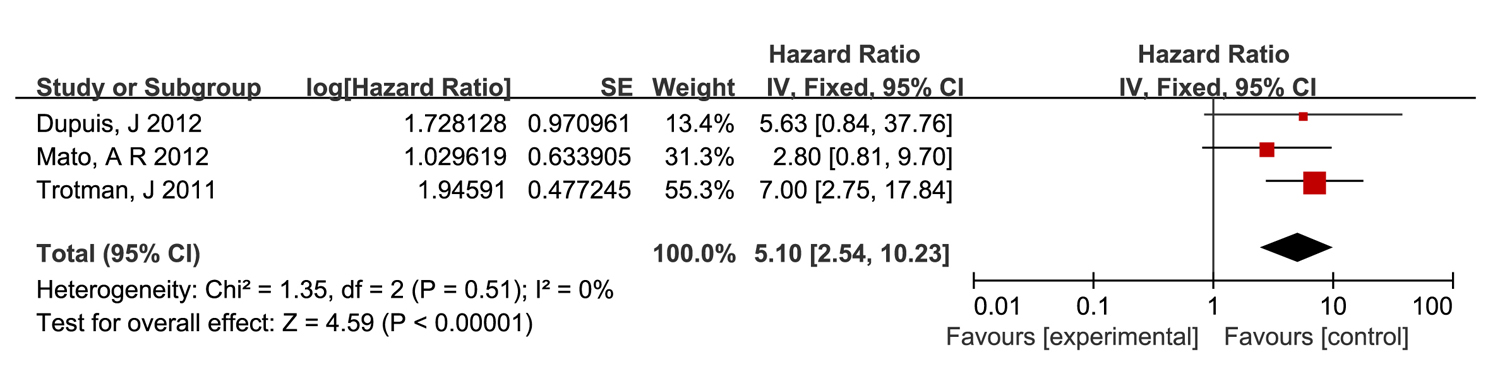
**

**Appendix**

The quality scale used in this study

Except when specified, the attributed value per item is 2 points if it is clearly defined in the article, 1 point if its description is incomplete or unclear, and 0 points if it is not defined or is inadequate.

Scientific design

(1) Study objective definition.

(2) Study design: prospective (2 points); retrospective (1 point); not defined (0 points).

(3) Outcome definition.

(4) Statistical considerations (fully reported with a preliminary assessment of the patient/sample number to be included and/or analyzed (2 points); patient/

sample number to be included and/or analyzed justified by the number of studied variables (minimum 10 patients per variable)(1 point); not defined (0 points).

(5) Statistical methods and tests description.

Generalizability

(1) Patient selection criteria, including histological type, disease stage, and treatment.

(2) Patients’ characteristics, including histology type,disease stage, and treatment.

(3) Initial workup.

(4) Treatment description.

(5) Number of ineligible patients with exclusion causes.

Results analysis

(1) Follow-up description, including the number of events.

(2) Survival analysis according to the standardized uptake value (SUV).

(3) Univariate analysis of the prognostic factors for survival: report of the relative risk with the confidence interval

(2 points); results without evaluation of the relative risk and its confidence interval (1 point); not reported or inadequate (0 points).

(4) Multivariate analysis of the prognostic factors for survival: report the relative risk with the confidence interval (2 points); results without evaluation of the

relative risk and its confidence interval (1 point); not reported or inadequate (0 points).

The PET reports

(1) Patients characteristics: weight/height; blood sugar level; histologic subtype.

(2) 18F-fluoro-2-deoxy-glucose-PET acquisition protocol characteristics: fasting duration; injected dose of 18F-fluoro-2-deoxy-glucose; delay between injection and data acquisition.

(3) Technical parameters: investigation area; delay between computed tomography thorax and PET acquisition; SUV formula; type of SUV; type of PET engine; duration of emission time; duration of transmission time; attenuation and reconstruction parameters.

(4) The analysis of the relationship between SUV was performed without knowledge of survival results and conversely (double blind).

(5) SUV cutoff definition.
